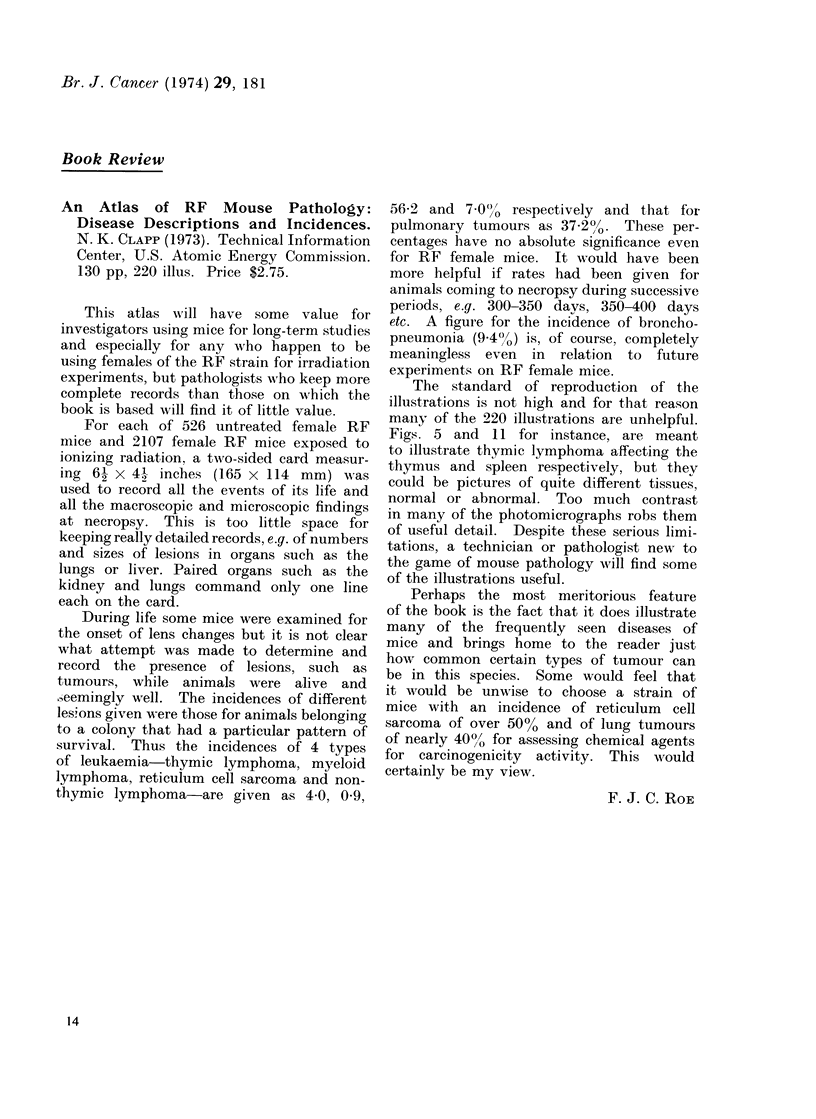# An Atlas of RF Mouse Pathology: Disease Descriptions and Incidences

**Published:** 1974-02

**Authors:** F. J. C. Roe


*Br. J. Cancer* (1974) **29**, 181

### *Book Review*

**An Atlas of RF Mouse Pathology: Disease Descriptions and Incidences.** N. K. Clapp (1973). Technical Information Center, U.S. Atomic Energy Commission. 130 pp, 220 illus. Price $2.75.

This atlas will have some value for investigators using mice for long-term studies and especially for any who happen to be using females of the RF strain for irradiation experiments, but pathologists who keep more complete records than those on which the book is based will find it of little value.

For each of 526 untreated female RF mice and 2107 female RF mice exposed to ionizing radiation, a two-sided card measuring $6\frac{1}{2} \times 4\frac{1}{2}$ inches (165 × 114 mm) was used to record all the events of its life and all the macroscopic and microscopic findings at necropsy. This is too little space for keeping really detailed records, *e.g.* of numbers and sizes of lesions in organs such as the lungs or liver. Paired organs such as the kidney and lungs command only one line each on the card.

During life some mice were examined for the onset of lens changes but it is not clear what attempt was made to determine and record the presence of lesions, such as tumours, while animals were alive and seemingly well. The incidences of different lesions given were those for animals belonging to a colony that had a particular pattern of survival. Thus the incidences of 4 types of leukaemia—thymic lymphoma, myeloid lymphoma, reticulum cell sarcoma and non-thymic lymphoma—are given as 4·0, 0·9, 56·2 and 7·0% respectively and that for pulmonary tumours as 37·2%. These percentages have no absolute significance even for RF female mice. It would have been more helpful if rates had been given for animals coming to necropsy during successive periods, *e.g.* 300–350 days, 350–400 days *etc.* A figure for the incidence of bronchopneumonia (9·4%) is, of course, completely meaningless even in relation to future experiments on RF female mice.

The standard of reproduction of the illustrations is not high and for that reason many of the 220 illustrations are unhelpful. Figs. 5 and 11 for instance, are meant to illustrate thymic lymphoma affecting the thymus and spleen respectively, but they could be pictures of quite different tissues, normal or abnormal. Too much contrast in many of the photomicrographs robs them of useful detail. Despite these serious limitations, a technician or pathologist new to the game of mouse pathology will find some of the illustrations useful.

Perhaps the most meritorious feature of the book is the fact that it does illustrate many of the frequently seen diseases of mice and brings home to the reader just how common certain types of tumour can be in this species. Some would feel that it would be unwise to choose a strain of mice with an incidence of reticulum cell sarcoma of over 50% and of lung tumours of nearly 40% for assessing chemical agents for carcinogenicity activity. This would certainly be my view.

F. J. C. Roe